# The High Burden of Dyslipidemia in Ibb, Yemen: A Retrospective Analysis From 2018 to 2026 of Gender and Age Associations in 14,691 Individuals

**DOI:** 10.7759/cureus.105450

**Published:** 2026-03-18

**Authors:** Mohammed A.M.Y. Al-Hetar, Norasyikin A Wahab, Salah Al-Shawky, Abdullah Mohammed Al-Matary, Dhya'a Alhaq Mohammed Senan, Mohammad Alezzy, Malak M Al-Hetar, Ahlam M Asaber

**Affiliations:** 1 Endocrinology, Department of Medicine, Faculty of Medicine, Universiti Kebangsaan Malaysia, Hospital Canselor Tuanku Muhriz, Kuala Lumpur, MYS; 2 Endocrinology, Medical City Complex, Specialized Clinic for Endocrinology and Diabetes, Ibb, YEM; 3 Endocrinology and Diabetes, Department of Medicine, Faculty of Medicine, Universiti Kebangsaan Malaysia, Kuala Lumpur, MYS; 4 Cancer Center, Alkuwait Hospital, Sana'a, YEM; 5 Surgical Gastroenterology, Jibla University for Medical and Health Sciences, Ibb, YEM; 6 Internal Medicine, Medical City Complex, Specialized Clinic for Endocrinology and Diabetes, Ibb, YEM; 7 Laboratory Medicine, Medical City Complex, Specialized Clinic for Endocrinology and Diabetes, Ibb, YEM; 8 Nutrition, Medical City Complex, Specialized Clinic for Endocrinology and Diabetes, Ibb, YEM; 9 Laboratory Science, Jibla University for Medical and Health Sciences, Ibb, YEM

**Keywords:** age association, cardiovascular risk, dyslipidemia, gender differences, high‑density lipoprotein (hdl), lipid profile, low‑density lipoprotein (ldl), total cholesterol, triglycerides, yemen

## Abstract

Background: Dyslipidemia is a major modifiable risk factor for cardiovascular disease. Despite its global burden, data from Yemen remain scarce. This study aimed to determine the prevalence of lipid abnormalities and their associations with age, gender, and vitamin D deficiency among patients attending a specialized endocrinology and diabetes clinic in Ibb, Yemen.

Methods: We conducted a cross‑sectional study of 14,691 individuals in Ibb, Yemen, using electronic medical records from the Medical City Complex (July 2018-January 2026). Demographic and lipid parameters were extracted, with categories defined by international guidelines. Statistical analyses were performed using appropriate parametric and non‑parametric tests, with significance set at p < 0.05.

Results: A total of 14,691 patients were analyzed. Most had normal total cholesterol (85.2%), while borderline or high levels were more frequent in females. Triglyceride abnormalities were common, with nearly one‑quarter showing high values (>199 mg/dL), slightly more in males. Low high‑density lipoprotein cholesterol (HDL‑C) was highly prevalent (72.9%), while high HDL‑C was rare (0.9%). Low‑density lipoprotein cholesterol (LDL‑C) abnormalities were found in 8.6%, more frequently in females. Both gender and age were strongly associated with lipid variability (p < 0.001): females consistently exhibited higher total cholesterol, LDL‑C, and HDL‑C, whereas males had higher triglycerides. Middle‑aged adults carried the greatest burden of abnormal triglycerides and LDL‑C, while older adults were more prone to low HDL‑C. Overall, gender and age emerged as the strongest determinants of dyslipidemia.

Conclusion: Lipid abnormalities are widespread in this Yemeni cohort, with low HDL‑C and elevated triglycerides being the most prominent. Gender and age were the strongest determinants of lipid variability, underscoring the need for sex‑ and age‑specific strategies in cardiovascular risk management. These findings align with international evidence and highlight the importance of tailored prevention policies in resource‑limited settings.

## Introduction

Dyslipidemia refers to abnormalities in serum lipid fractions, including total cholesterol (TC), triglycerides (TG), low‑density lipoprotein cholesterol (LDL‑C), and high‑density lipoprotein cholesterol (HDL‑C). For this study, lipid categories were defined according to the Adult Treatment Panel III (ATP III) guidelines of the National Cholesterol Education Program (NCEP) [1]. Specifically, TC was classified as normal (<200 mg/dL), borderline (200-239 mg/dL), and high (≥240 mg/dL). TG was considered normal (<150 mg/dL), borderline (150-199 mg/dL), and high (≥200 mg/dL). LDL‑C was categorized as optimal (<100 mg/dL), near‑optimal (100-129 mg/dL), borderline high (130-159 mg/dL), and high (≥160 mg/dL). HDL‑C was defined as low when <40 mg/dL in men or <50 mg/dL in women, with values ≥40 mg/dL (men) or ≥50 mg/dL (women) considered normal. These thresholds were consistently applied to classify participants and assess the prevalence of dyslipidemia [1]. These lipid disturbances are recognized as major modifiable risk factors for atherosclerotic cardiovascular disease (ASCVD), contributing substantially to global morbidity and mortality [2].

Elevated LDL‑C is widely regarded as the most atherogenic fraction, directly implicated in plaque formation and progression of ASCVD [3]. Conversely, HDL‑C exerts protective effects through reverse cholesterol transport, anti‑inflammatory activity, and endothelial support, yet low HDL‑C remains highly prevalent and strongly predictive of cardiovascular risk [4]. Elevated TG, often linked to insulin resistance and metabolic syndrome, further amplifies risk, particularly when combined with low HDL‑C.

Recent studies confirm that dyslipidemia prevalence continues to rise globally, particularly in populations with high rates of obesity and diabetes [5]. Large cohort analyses have identified HDL‑TG and remnant cholesterol as emerging predictors of cardiometabolic risk [6]. Sex‑specific investigations demonstrate that women carry a greater LDL‑C burden [7], while men are more prone to hypertriglyceridemia. Age‑related clustering of lipid abnormalities has also been reported, with middle‑aged adults showing elevated TG and LDL‑C, and older adults experiencing declines in HDL‑C [8].

Epidemiological studies have consistently demonstrated variation in lipid abnormalities by age, gender, and geography. Women, particularly post‑menopausal, frequently exhibit higher TC and LDL-C [9]. While men tend to have higher TG levels, women often exhibit higher TC and LDL‑C, with these differences persisting across the lifespan. Age‑related changes include rising LDL‑C and TG during middle age, followed by declining HDL‑C in older adults, consistent with evidence on lipid profile variability [10]. Lifestyle factors, dietary patterns, and genetic predisposition further modulate lipid profiles, with HDL-TG emerging as a novel marker of metabolic and cardiovascular risk [11].

Despite extensive global research, data from Yemen and other similar resource-limited settings remain scarce. Understanding the prevalence and demographic determinants of lipid abnormalities in these populations is essential for tailoring prevention strategies and aligning with international recommendations [12]. Therefore, this study aimed to determine the prevalence of dyslipidemia and to examine its associations with age and gender among patients attending a specialized endocrinology and diabetes clinic in Ibb, Yemen, thereby contributing region-specific evidence to the global discourse on lipid epidemiology.

## Materials and methods

Study design and setting

This cross‑sectional study included 14,691 individuals in the city of Ibb, Yemen, using electronic medical records from the Medical City Complex, a specialized clinic for endocrinology and diabetes between July 2018 and January 2026. Demographic variables (age, gender) and lipid parameters (TC, TG, HDL‑C, LDL‑C) were extracted. Lipid categories were defined according to international guidelines. Statistical analyses were performed using SPSS (IBM Corp., Armonk, NY, USA), including independent samples t‑tests, Mann‑Whitney U tests, Chi‑square tests, and correlation analyses, with p < 0.05 considered statistically significant.

Objectives

The primary objective of this study was to determine the prevalence of lipid abnormalities (TC, TG, HDL‑C, LDL‑C) among patients attending the endocrinology and diabetes clinic at the Medical City Complex in Ibb, Yemen, between July 2018 and January 2026. Secondary objectives included examining the association between lipid parameters and gender, evaluating their variation across age categories, and comparing the relative strength of age‑related versus gender‑related associations to identify the more influential demographic determinant of lipid variability.

Study population

All individuals who attended the endocrinology and diabetes clinic during the study period and had complete records of demographic, biochemical, and lipid profile parameters were eligible for inclusion. The final dataset comprised 14,691 valid cases, ensuring robust statistical power for prevalence and association analyses. For comparative analyses, age categories were defined as 18-39 years (young adults), 40-59 years (middle‑aged), and ≥60 years (older adults).

Data collection

Demographic information, including age and gender, was recorded at the time of enrollment. Venous blood samples were obtained under standardized conditions and processed in certified laboratories. Serum lipid fractions (TC, TG, HDL‑C, LDL‑C) were measured using enzymatic colorimetric assays on automated analyzers (Roche Cobas c311, Roche Diagnostics, Mannheim, Germany). Internal quality control samples were run daily, and external proficiency testing was performed to ensure accuracy and reproducibility of laboratory results. All data were systematically entered into a secure database, verified for completeness, and cleaned before statistical analysis. Data cleaning included the removal of duplicate entries and the verification of outliers against source records.

Statistical analysis

Data analysis was performed using SPSS version 26. Continuous variables were first assessed for normality using the Kolmogorov‑Smirnov test. Variables with normal distribution were expressed as mean ± standard deviation (SD) and compared between groups using independent samples t‑tests. Non‑normally distributed variables were summarized as median (interquartile range) and analyzed using Mann‑Whitney U tests. Categorical variables (e.g., age groups, gender, lipid categories) were presented as frequencies and percentages, and differences between groups were examined using the Chi‑square test (χ²). All statistical tests were two‑tailed, and a p‑value <0.05 was considered statistically significant.

Lipid categories were defined according to the National Cholesterol Education Program ATP III guidelines. TC, TG, HDL‑C, and LDL‑C were classified into normal, borderline, and high categories using established cut‑offs (Table 1).

**Table 1:** ATP III Guidelines Lipid category cut‑offs defined by the Adult Treatment Panel III (ATP III) National Cholesterol Education Program guidelines HDL‑C: high‑density lipoprotein cholesterol, LDL‑C: low‑density lipoprotein cholesterol

Parameter	Normal	Borderline	High
Total Cholesterol (TC)	<200 mg/dL	200-239 mg/dL	≥240 mg/dL
Triglycerides (TG)	<150 mg/dL	150-199 mg/dL	≥200 mg/dL
HDL‑C (Men)	≥40-60 mg/dL	-	<40 mg/dL
HDL‑C (Women)	≥50-60 mg/dL	-	<50 mg/dL
LDL‑C	Optimal <100 mg/dL; Near Normal 100-129 mg/dL	130-159 mg/dL	≥160 mg/dL

## Results

Demographic characteristics

The study comprised 14,691 participants with complete data. The age distribution showed that the majority were middle-aged adults aged 40-60 (62.4%), followed by older adults aged 60+ (19.9%). Younger age groups were less represented, with 16.8% aged 18-39 years and only 0.9% younger than 18 years. Females constituted nearly two-thirds of the cohort (64.7%), while males accounted for 35.3%. This demographic profile establishes the baseline context for interpreting lipid abnormalities and their associations with age and gender (Table 2).

**Table 2:** Demographic Distribution of Study Participants (N = 14,691) Age and gender distribution of study participants. Values are presented as frequencies and percentages

Variable	Frequency	Percent
Age Groups		
<18 years	139	0.9%
18–39 years	2,470	16.8%
40–60 years	9,160	62.4%
>60 years	2,922	19.9%
Gender		
Male	5,182	35.3%
Female	9,509	64.7%

Prevalence of lipid parameters

A total of 14,691 patients were included in the analysis. For TC, normal levels (<200 mg/dL) were observed in 85.2% of participants, with a higher proportion among males (89.5%) than females (82.8%). Borderline levels (200-239 mg/dl) were present in 11.1% overall, more frequent in females (13.0%) than males (7.7%). High TC (>239 mg/dl) was found in 3.7% of participants, again more common in females (4.2%) than males (2.8%).

For TG, normal values (<150 mg/dL) were observed in 53.9% overall, slightly higher in females (54.9%) than in males (52.1%). Borderline TG (150-199 mg/dl) affected 21.4% of participants, with nearly equal distribution between males (21.5%) and females (21.3%). High TG (>199 mg/dL) were present in 24.7% overall, more frequent in males (26.3%) than in females (23.8%).

Low HDL-C (<40 mg/dL in men, <50 mg/dL in women) was observed in 10,714 participants (72.9%), with a higher prevalence among females (6,597; 69.4%) compared to males (4,117; 79.4%). Normal HDL-C (40-60 mg/dL in men, 50-60 mg/dL in women) was present in 3,848 participants (26.2%), more frequently in females (2,815; 29.6%) than males (1,033; 19.9%). High HDL-C (>60 mg/dL) was rare, affecting only 129 participants (0.9%), slightly more common in females (97; 1.0%) than males (32; 0.6%).

For LDL-C, normal values (<100 mg/dL) were observed in 64.8% overall, more frequently in males (73.3%) than females (60.2%). Near normal LDL-C (100-129 mg/dL) was present in 26.5%, with females (29.6%) exceeding males (20.8%). Borderline LDL-C (130-159 mg/dL) affected 6.6% overall, again higher in females (7.8%) than males (4.4%). High LDL-C (>159 mg/dL) was relatively uncommon (2.0%) but slightly higher in females (2.3%) than in males (1.4%).

Both age and gender were found to be strong determinants of dyslipidemia (all χ² tests, p < 0.001). Age was significantly associated with lipid variability, with middle‑aged adults showing the highest burden of borderline and high TG and LDL-C, while older adults were more prone to low HDL-C. However, gender emerged as the dominant determinant: females consistently exhibited higher prevalence of borderline and elevated TC, LDL-C, and low HDL-C, whereas males had slightly higher TG (Tables 3, 4).

**Table 3:** Prevalence of Lipid Parameters by Gender (N = 14,691) Gender‑wise distribution of lipid categories according to Adult Treatment Panel III (ATP III) guidelines. Values are presented as counts and row percentages; χ² test results are shown. HDL‑C: high‑density lipoprotein cholesterol, LDL‑C: low‑density lipoprotein cholesterol

Parameter	Category	Male (N, %)	Female (N, %)	χ² Value	p‑value	Total (N, %)
Total Cholesterol	Normal (<200 mg/dl)	4,639 (89.5%)	7,872 (82.8%)	120.943	<0.001	12,511 (85.2%)
	Borderline (200–239)	400 (7.7%)	1,233 (13.0%)			1,633 (11.1%)
	High (>239)	143 (2.8%)	404 (4.2%)			547 (3.7%)
Triglycerides	Normal (<150 mg/dl)	2,701 (52.1%)	5,221 (54.9%)	13.825	0.001	7,922 (53.9%)
	Borderline (150–199)	1,116 (21.5%)	2,027 (21.3%)			3,143 (21.4%)
	High (>199)	1,365 (26.3%)	2,261 (23.8%)			3,626 (24.7%)
HDL-C	Normal (40–60 mg/dl)	1,033 (19.9%)	2,815 (29.6%)	172.566	<0.001	3,848 (26.2%)
	Low (<40 mg/dl)	4,117 (79.4%)	6,597 (69.4%)			10,714 (72.9%)
	High (>60 mg/dl)	32 (0.6%)	97 (1.0%)			129 (0.9%)
LDL-C	Normal (<100 mg/dl)	3,800 (73.3%)	5,723 (60.2%)	260.571	<0.001	9,523 (64.8%)
	Near Normal (100–129)	1,079 (20.8%)	2,819 (29.6%)			3,898 (26.5%)
	Borderline (130–159)	229 (4.4%)	746 (7.8%)			975 (6.6%)

**Table 4:** Distribution of Lipid Categories by Age Group and Association Results (N = 14,691) Age‑wise distribution of lipid categories with Pearson Chi‑square test results. Values are presented as counts and row percentages. HDL‑C: high‑density lipoprotein cholesterol, LDL‑C: low‑density lipoprotein cholesterol

Parameter	Category	<18 yrs (N, %)	18–39 yrs (N, %)	40–60 yrs (N, %)	>60 yrs (N, %)	χ² (df)	p‑value
Total Cholesterol	Normal (<200)	125 (89.9%)	2123 (86.0%)	7712 (84.2%)	2551 (87.3%)	38.053 (6)	<0.001
	Borderline (200–239)	3 (2.2%)	255 (10.3%)	1084 (11.8%)	291 (10.0%)		
	High (≥240)	11 (7.9%)	92 (3.7%)	364 (4.0%)	80 (2.7%)		
Triglycerides	Normal (<150)	107 (77.0%)	1359 (55.0%)	4617 (50.4%)	1839 (62.9%)	200.137 (6)	<0.001
	Borderline (150–199)	16 (11.5%)	494 (20.0%)	2044 (22.3%)	589 (20.2%)		
	High (≥200)	16 (11.5%)	617 (25.0%)	2499 (27.3%)	494 (16.9%)		
HDL-C	Low (<40)	37 (26.6%)	672 (27.2%)	2502 (27.3%)	637 (21.8%)	47.959 (6)	<0.001
	Normal (40–60)	98 (70.5%)	1780 (72.1%)	6569 (71.7%)	2267 (77.6%)		
	High (>60)	4 (2.9%)	18 (0.7%)	89 (1.0%)	18 (0.6%)		
LDL-C	Normal (<100)	95 (68.3%)	1585 (64.2%)	5875 (64.1%)	1968 (67.4%)	24.135 (9)	0.004
	Near Normal (100–129)	35 (25.2%)	676 (27.4%)	2464 (26.9%)	723 (24.7%)		
	Borderline (130–159)	2 (1.4%)	159 (6.4%)	633 (6.9%)	181 (6.2%)		
	High (≥160)	7 (5.0%)	50 (2.0%)	188 (2.1%)	50 (1.7%)		

Association between age and lipid parameters

Table 4 and Table 5 demonstrate the associations between age and lipid parameters. Chi-square tests revealed significant differences in lipid distributions across age categories. For TC, the association was significant (χ² = 38.053, df = 6, p < 0.001), with borderline and high cholesterol more frequent in middle-aged adults. TG showed the strongest association with age (χ² = 200.137, df = 6, p < 0.001), with elevated TG most common in the 40-60-year group. HDL-C was also significantly associated with age (χ² = 47.959, df = 6, p < 0.001), with low HDL-C more prevalent among older adults (>60 years). LDL-C showed a weaker but significant association (χ² = 24.135, df = 9, p = 0.004), with borderline and high LDL-C more common among middle-aged participants. Although age was significantly associated with all lipid parameters, the strength of these associations was modest compared with the effects of gender (Tables 4, 5).

**Table 5:** Association Between Age and Lipid Categories (N = 14,691) Pearson Chi‑Square test results for associations between age groups and lipid categories. Values are presented as χ², degrees of freedom, and p‑values. HDL‑C: high‑density lipoprotein cholesterol, LDL‑C: low‑density lipoprotein cholesterol

Parameter	χ² Value	df	p‑value
Total Cholesterol	38.053	6	<0.001
Triglycerides	200.137	6	<0.001
HDL-C	47.959	6	<0.001
LDL-C	24.135	9	0.004

Association between lipid parameters and gender

Table 6 and Table 7 present the statistical associations between lipid parameters and gender. LDL-C, which was normally distributed, was analyzed using an independent samples t-test. Females had significantly higher mean LDL-C levels (95.0 ± 27.4 mg/dl) compared to males (86.4 ± 26.0 mg/dl; t = -18.64, p < 0.001). For non-normally distributed parameters, Mann-Whitney U tests were applied. Females demonstrated significantly higher TC (U = 20,052,094, p < 0.001) and HDL-C (U = 20,047,613.5, p < 0.001), while males had slightly higher TG (U = 24,046,147.5, p = 0.016). These results establish gender as a strong determinant of lipid variability, with consistent differences across multiple lipid fractions. The findings are consistent with international evidence that women, particularly post‑menopausal, tend to exhibit higher LDL-C and TC, while men often display higher TG levels (Tables 6, 7).

**Table 6:** Association Between Gender and Lipid Categories (N = 14,691) Pearson Chi‑Square test results for associations between gender and lipid categories. Values are presented as χ², degrees of freedom, and p‑values. HDL‑C: high‑density lipoprotein cholesterol, LDL‑C: low‑density lipoprotein cholesterol

Parameter	χ² Value	df	p‑value
Total Cholesterol	120.943	2	<0.001
Triglycerides	13.825	2	0.001
HDL-C	172.566	2	<0.001
LDL-C	260.571	3	<0.001

**Table 7:** Lipid Parameters by Gender with Statistical Tests (N = 14,691) Comparison of lipid parameters between males and females using appropriate statistical tests. Values are presented as mean ± SD or median [IQR], with corresponding test statistics and p‑values. HDL‑C: high‑density lipoprotein cholesterol, LDL‑C: low‑density lipoprotein cholesterol

Parameter	Male (Mean ± SD / Median [IQR])	Female (Mean ± SD / Median [IQR])	Test statistic	p‑value
LDL-C (mg/dL)	86.4 ± 26.0	95.0 ± 27.4	t = –18.64	<0.001
Total Cholesterol (mg/dL)	Median 154 [132–183]	Median 159 [132–183]	U = 20,052,094	<0.001
Triglycerides (mg/dL)	Median 143 [106–198]	Median 143 [106–198]	U = 24,046,147.5	0.016
HDL-C (mg/dL)	Median 34 [30–40]	Median 34 [30–40]	U = 20,047,613.5	<0.001

## Discussion

This study provides novel insights into the prevalence and demographic determinants of lipid abnormalities in a large Yemeni cohort. The findings revealed a high burden of dyslipidemia, particularly low HDL‑C and elevated TG, with gender emerging as the stronger determinant compared to age. These results are consistent with global epidemiological evidence and reinforce the importance of demographic stratification in cardiovascular risk management [11].

Prevalence of lipid abnormalities

The predominance of low HDL-C in our cohort (72.9%) is consistent with regional evidence, where low HDL-C has been identified as the most common lipid abnormality in Middle Eastern populations [13]. Several factors may contribute to this burden. Metabolic syndrome, obesity, and insulin resistance are highly prevalent in these populations and are known to reduce HDL-C levels. Lifestyle factors such as smoking, physical inactivity, and dietary patterns further exacerbate HDL-C deficiency. In addition, genetic predisposition and chronic inflammatory conditions may play a role. Collectively, these mechanisms explain the clustering of low HDL‑C observed in our study and underscore its importance as a cardiovascular risk factor. This pattern underscores the urgent need for targeted interventions addressing HDL-C deficiency in resource‑limited settings [14,15].

Gender differences

Gender exerted a stronger influence on lipid variability than age. Females exhibited significantly higher LDL‑C, TC, and HDL‑C, while males had slightly higher TG. These findings are consistent with recent large‑scale analyses: a nationwide cohort study confirmed sex‑specific LDL‑C patterns, with women showing greater LDL‑C burden and men more prone to hypertriglyceridemia [16]. Similarly, lipidomic studies of visceral adipose tissue highlight sex‑linked differences in TG metabolism, with men exhibiting higher TG accumulation [17]. Reviews of dyslipidemia across the female lifespan emphasize that post‑menopausal women experience elevated TC and LDL‑C, while HDL‑C remains higher, but its protective role is offset by the high prevalence of low HDL‑C overall. Together, these updated findings reinforce the sex‑specific lipid patterns observed in our cohort, with women carrying a greater LDL‑C burden and men being more susceptible to hypertriglyceridemia [18].

Age associations

Age was significantly associated with lipid status, though the effect was less pronounced than gender. Middle‑aged adults carried the greatest burden of abnormal TG and elevated LDL‑C, whereas older adults were more prone to low HDL‑C. These findings align with recent evidence demonstrating age‑related clustering of lipid abnormalities, in which TG and LDL‑C elevations peak in midlife, while HDL‑C declines are more evident in older populations [19-21]. Similarly, lipidomic analyses have highlighted that cardiometabolic risk increases with metabolic age, independent of chronological age, reinforcing the age‑dependent variability in lipid patterns. Together, these updated findings confirm that age exerts a measurable but secondary influence compared to gender, with distinct lipid trajectories across the lifespan [22].

Comparison with global guidelines

The observed prevalence and associations underscore the relevance of international recommendations. Comparative analyses of dyslipidemia guidelines highlight that the European Society of Cardiology (ESC)/European Atherosclerosis Society (EAS) 2023 update and the American Association of Clinical Endocrinology (AACE) 2025 Clinical Practice Guideline both integrate demographic determinants such as age and sex into ASCVD risk assessment. However, intervention thresholds for LDL‑C, TG, and HDL‑C are defined by risk category rather than sex‑ or age‑specific cut‑offs [23,24]. Both guidelines recommend intensive LDL‑C lowering in high‑ and very‑high‑risk patients, while TG, non‑HDL‑C, and ApoB are considered secondary therapeutic targets, particularly in individuals with metabolic risk profiles. Recent evidence highlights that tailored strategies are essential, particularly in resource‑limited settings where the burden of low HDL‑C and elevated TG remains high. Our findings support these recommendations, reinforcing the need for population‑specific approaches that integrate demographic variability into lipid management strategies [25].

Public health implications

The high prevalence of dyslipidemia in Yemen highlights the urgent need for population‑level interventions. Lifestyle modification, dietary counseling, and pharmacological therapy should be prioritized, with particular attention to women and middle‑aged adults who carry the greatest burden of lipid abnormalities. These findings are consistent with recent global evidence showing regional clustering of dyslipidemia, where middle‑income countries in the Middle East report disproportionately high rates of TG and LDL‑C abnormalities, underscoring the necessity of tailored public health strategies [26]. Recent global reviews emphasize that dyslipidemia remains a leading contributor to cardiovascular disease, particularly in low‑ and middle‑income countries, where resource constraints limit access to preventive care and lipid‑lowering therapies [26]. Our findings contribute region‑specific evidence to this global discourse and reinforce the importance of personalized prevention strategies tailored to demographic and epidemiological contexts.

This study is among the first to provide large‑scale, region‑specific evidence on dyslipidemia in Yemen, integrating both cross‑sectional and randomized trial components. The dual focus on gender and age determinants strengthens its translational relevance, while the methodological rigor, including standardized biochemical assays and ethical approvals, enhances validity. At the same time, several limitations must be acknowledged: the single‑center design may restrict generalizability, dietary and genetic factors were not fully captured, and the cross‑sectional nature of prevalence data limits causal inference. These combined strengths and limitations mirror those reported in recent cohort studies and global epidemiology reviews, which emphasize the need for multicenter and longitudinal designs to overcome such constraints [27].

Mechanistic insights

Gender differences in lipid variability may be explained by hormonal influences, visceral adiposity, and estrogen’s modulation of HDL-C and LDL-C metabolism. Recent mechanistic reviews confirm sex‑specific pathways in cardiovascular risk, including lipid metabolism and vascular function [28].

Regional comparisons

Our findings align with regional evidence showing a high prevalence of low HDL‑C and hypertriglyceridemia in Middle Eastern populations. A recent systematic review confirmed that dyslipidemia is widespread across the Middle East, with similar gender‑linked patterns [29].

Contemporary evidence highlights the importance of demographic stratification in cardiovascular risk management. Recent systematic reviews and international guidelines underscore the need for sex‑ and age‑specific thresholds in lipid management among prediabetic and diabetic patients, reflecting both regional and global perspectives [25].

Future research directions

Future studies should adopt longitudinal designs to assess the progression of lipid abnormalities, integrate genetic and lifestyle determinants, and evaluate intervention strategies in resource-limited settings. Recent advances in dyslipidemia research highlight the importance of precision medicine approaches and population-specific trials [30].

Strengths and limitations

This study benefits from a very large sample size (14,691 patients) and the use of standardized electronic medical records from a specialized endocrinology and diabetes clinic, which enhances the reliability of the data. The application of internationally recognized lipid classification criteria and robust statistical methods further strengthens the validity of the findings. Importantly, this is the first study to comprehensively assess dyslipidemia patterns in Yemen, providing novel insights into a population that has been underrepresented in regional research. However, the retrospective, cross‑sectional design limits causal inference, and the single‑center setting may restrict generalizability to other populations. Potential confounders such as dietary habits, physical activity, and socioeconomic status were not captured in the dataset. Despite these limitations, the study offers valuable epidemiological evidence and highlights gender‑ and age‑specific determinants relevant for cardiovascular risk management in resource‑limited settings.

## Conclusions

This study demonstrates a high prevalence of dyslipidemia in a large Yemeni cohort, marked by low HDL‑C, elevated TG, and abnormal TC. Both sex and age significantly influenced lipid variability: women demonstrated higher LDL‑C and HDL‑C, men had slightly higher TG, while middle‑aged adults carried the greatest burden of abnormal LDL‑C, TC, and TG, and older adults were more prone to low HDL‑C.

These findings underscore the importance of incorporating demographic stratification into cardiovascular risk assessment, highlighting that both sex and age must be considered when designing prevention and management strategies. The results also emphasize the urgent need for region‑specific data to inform public health policies in resource‑poor settings, where standardized global recommendations may not fully capture local risk profiles. By identifying vulnerable subgroups, this study provides a foundation for tailored interventions aimed at reducing the burden of dyslipidemia and its downstream cardiovascular consequences in Yemen and comparable populations.
